# Association between quality and duration of sleep and subjective cognitive decline: a cross-sectional study in South Korea

**DOI:** 10.1038/s41598-021-96453-x

**Published:** 2021-08-20

**Authors:** Hye Jin Joo, Jae Hong Joo, Junhyun Kwon, Bich Na Jang, Eun-Cheol Park

**Affiliations:** 1 Department of Public Health, Graduate School, Yonsei University, Seoul, Republic of Korea; 2 Institute of Health Services Research, Yonsei University, Seoul, Republic of Korea; 3 Department of Preventive Medicine, Yonsei University College of Medicine, 50 Yonsei-ro, Seodaemun-gu, Seoul, 03722 Republic of Korea

**Keywords:** Neurology, Risk factors

## Abstract

Sleep is being emphasized as a factor that improves mental health and quality of life. Here, we aimed to investigate the association between the quality and duration of sleep and subjective cognitive decline in the Korean population. We used the 2018 Korean Community Health Survey data that are nationwide representative data collected by the Korea Centers for Disease Control and Prevention. Sleep quality was measured using the Korean version of Pittsburgh Sleep Quality Index. The study population comprised 206,719 individuals aged 19 years and over. We used multiple logistic regression for the analysis. Individuals of both sexes with poor sleep quality were more likely to experience subjective cognitive decline compared with the reference group (good sleep quality) (men, odds ratio (OR) = 1.97 [95% confidence interval (CI) 1.86–2.09]; women, OR = 1.75 [95% CI 1.67–1.84]). U-shape associations were found between sleep duration and subjective cognitive decline. Additionally, the presence of depressive symptom or stress and health-related behaviors, including smoking, drinking, and not walking, were high-risk factors for subjective cognitive decline. Our results indicate that poor sleep quality might contribute to subjective cognitive decline in the Korean population. We suggest the implementation of intervention measures for poor sleep behaviors to prevent cognitive decline.

## Introduction

Cognitive decline is a natural change that occurs with aging and does not significantly interfere with the activities of daily life; however, it can also be an indication for the development of geriatric neurodegenerative diseases, such as dementia. Some individuals with mild cognitive impairment appear to remain stable or return to a healthy cognitive state over time, but in more than half the cases, this condition progresses to dementia within 5 years. Cognitive impairment involving memory complaints and deficits has been consistently shown to have a high risk of progressing to dementia, particularly of the Alzheimer type^1^. According to a previous study, a steeper decline in cognition in individuals with new infarctions increases the risk of the development of additional infarctions, which may also contribute to the development of dementia^2^. Dementia is one of the most common diseases worldwide and the greatest global challenge related to health and society. The number of people with dementia is rising rapidly and is expected to increase from around 47 million in 2015 to 66 million in 2030 and to 131 million in 2050. Because dementia, unlike mild cognitive impairment, affects an individual’s everyday life or social functioning, it is a heavy burden on those with the disease, their families, and society^3^. An individual's subjective cognitive decline (SCD) is considered an early symptom of dementia^4^. Thus, the identification of cognitive decline could lead to secondary prevention through the implementation of measures to control risk factors. Dementia is no longer considered an inevitable consequence of aging and can be prevented or treated. Lifestyle-related factors might reduce or increase an individual's risk of developing dementia^3^.

Sleep affects a number of psychological functions, including cognitive function. Good sleep quality is a well-recognized predictor of physical and mental health, wellness, and overall vitality. Healthy sleep behavior has been known to be a factor that improves quality of life as well as mental health. Sleep disturbance and poor sleep quality might be caused by one or more of the following factors: physical health conditions, side effects of medications, other aspects of physical illness, and neurodegenerative changes; further, they can also be related to psychiatric disorders, such as depression, anxiety, and schizophrenia^3,5^. In modern society, many people do not sleep enough to accommodate changes in their daily schedules or to prioritize other activities. Insufficient sleep may have adverse effects on cardiovascular, endocrine, and immune functions, in addition to having a negative effect on mood^6–9^. Further, studies have reported that both short and long sleep durations increase the total mortality risk^10–12^. The standardized measures of sleep quality are the Karolinska Sleep Diary^13^, Verran and Snyder-Halpern Sleep Scale^14^, and Pittsburgh Sleep Quality Index (PSQI)^15^, of which the PSQI is the most widely used^5,16–18^. PSQI was originally designed for use in clinical populations as a simple and valid measure for assessing both sleep quality and disturbances that might affect sleep quality^15^.

As the importance of sleep quality as well as of sleep duration has become more evident, recent sleep studies have begun to focus more on sleep quality. In addition, there is a growing concern regarding sleeping habits and sleep disturbances in Asian populations, including in those residing in Korea and Japan^19^. Although many studies on sleep duration have been actively conducted in a meantime, an insufficient number of studies about sleep quality have been performed in the Korean population. Unlike previous study in Korean population, this study considered sleep time along with the PSQI index and performed analysis by gender^20^. Accordingly, we aimed to comprehensively examine how sleep quality and sleep duration are related to subjective cognitive decline in the Korean population. We hypothesized that poor sleep quality and short or long sleep durations would be associated with the risk of cognitive decline. Therefore, the purpose of this study was to investigate the link between quality and duration of sleep and cognitive function in the Korean population.

## Materials and methods

### Data collection and study population

Data were derived from the 2018 Korea Community Health Survey (KCHS), a constitutes community-based, nationwide representative data. KCHS was designed by the Korea Centers for Disease Control and Prevention (KCDC) to assess the efficacy of community-level health promotion and disease prevention programs since 2008^21^. KCHS has been conducted annually at national public health centers. The surveyed subjects are aged 19 years or older and are selected by the sampling method. KCHS is annually reviewed and approved by the institutional review board of the KCDC, and written informed consent is obtained from all the participants. In 2018, KCHS conducted a nationwide sleep quality survey using the Pittsburgh Sleep Quality Index for the first time in Korea. The initial study population comprised 228,340 individuals. Among them, we excluded the following participants who did not respond to the questions or those whose data contained missing values for variables: no record of subjective cognitive decline (n = 167); no record of sleep quality (n = 11,428); no record of PHQ-9 score (n = 411); no record of health-related behavior (n = 186); no record of stress (n = 87); no record of marriage status (n = 276); no record of educational level (n = 356); no record of occupation (n = 964); no record of income (n = 7,746). Finally, 206,719 participants (91,805 male and 114,914 female) were used as a sample in the study.

### Subjective cognitive decline

The main objective of this study was to analyze subjective cognitive decline. SCD was self-observed impairment of more frequent or worsening of memory loss or confusion within the prior 12 months. The questionnaire on cognitive function includes questions regarding subjective cognitive decline. For example, the survey question for SCD is as follows: “During the last year, have you experienced memory loss or confusion getting worse or happening more often?” The response categories are classified as “yes” or “no.”

### Sleep quality and duration

The main exposures of interest were sleep quality and duration. Sleep quality was measured using the Korean version of Pittsburgh Sleep Quality Index (PSQI-K)^22^. PSQI-K is a self-reported questionnaire that measures the quality and patterns of sleep over a period of a month. It contains 19 items and seven sleep components: subjective sleep quality, sleep latency, sleep duration, habitual sleep efficiency, sleep disturbance, use of sleep medication, and daytime dysfunction. Each component is scored on a scale ranging from 0 to 3. The global PSQI score is calculated by adding the scores of all the components together and it ranges from 0 to 21. Scores of greater than 5 are generally used to indicate poor sleep quality. Thus, in this study, we classified the survey participants into two groups as follows: the good sleep quality (scores of 0–5) and poor sleep quality (scores of 6–21) groups. As a reference, we also validated that previous studies on the Korean population also used a score of 5 as the cutoff point^17,23^. Sleep duration was measured by evaluating the responses to the following question: “How much sleep do you have per day?” Further, the questionnaire was designed for the participants to respond in hours and minutes. For the analysis, we classified the participants based on the hours of sleep as follows: < 5, 5 ≤ to < 6, 6 ≤ to < 7, 7 ≤ to < 8, 8 ≤ to < 9, and 9 ≤ h. According to World Sleep Society, the optimal sleep duration is about 7 to 8 hours^24^. Therefore, we set the individuals with the optimal sleep duration, ≤ 7 to < 8 h, were assigned to the reference group.

### Covariates

In this study, demographic, socioeconomic, and health-related characteristics were included as covariates in the fully adjusted models. The demographic and socioeconomic covariates included age, marital status (married, divorced, separated, or widowed, unmarried), educational level (high school or below, college, graduate school or above), region (metropolitan, urban, rural), household income (low, medium–low, medium–high, high), and job type (specialized job, office worker, sales and service, agriculture and fishery, manual worker, others). The household income groups were divided into quartiles using the monthly average household income. The health-related covariates included depression, stress, health-related behaviors, and body mass index (underweight, normal, overweight, obese). Depressive symptom was measured using the Patient Health Questionnaire-9 (PHQ-9) and was classified as “yes” (PHQ-9 score ≥ 10) and “no” (PHQ-9 score < 10). The level of stress was determined using the following question “How much stress do you feel in your daily life?” Response categories were classified as “yes” (very much or much) and “no” (a little or rarely). The health-related behaviors that were analyzed included the current smoking status, high-risk alcohol use, and engaging in walking/physical activity for more than 30 min per day for more than 5 days in the last week. Individuals who did not currently smoke, who did not engage in high-risk alcohol consumption, and who had walked more than 30 min per day for more than 5 days in the last week were classified as “yes,” indicating that they had a healthy lifestyle.

### Ethical standards

Korea Community Health Survey data provides anonymous, secondary data that is publicly available for scientific use. A more detailed description of the KCHS can be found on the website (https://chs.kdca.go.kr/).

### Statistical analysis

Due to gender differences in sleep quality, all analyzes were stratified by sex^25^. All analyses included the use of sampling weighted variables constructed by the KCHS.weighted variables. A descriptive analysis was performed to examine the distribution of the general characteristics of the study population. We calculated the frequency and percentages of each variable and performed chi-square tests to examine significant differences in cognitive function depending on sleep quality and duration. The statistical significance level was defined as a p-value of < 0.05. A multiple logistic regression analysis was performed to determine odds ratios (ORs) and 95% confidence intervals (CIs) to identify the association between sleep quality, duration, and cognitive function after adjusting for sociodemographic and health-related covariates. ORs and 95% CIs were calculated to compare parameters between those who had good sleep behavior and those who had poor sleep behavior. A subgroup analysis of depression, stress, and health-related behavior was performed based on sleep quality and cognitive function. All the statistical analyses were performed using the SAS 9.4 software (SAS Institute, Cary, NC, USA).

### Ethical approval

Korea Community Health Survey (KCHS) data is publicly accessible and written informed consent is obtained from all the participants before participating in the survey. Instruments and study processes used for the study were approved by the Korea Centers for Disease and Control and Prevention Institutional Review Board. Respondents’ information was completely anonymized for use for research purposes. The authors assert that all procedures contributing to this work comply with the ethical standards of the relevant national and institutional committees on human experimentation and with the Helsinki Declaration of 1975, as revised in 2000.

## Results

The general characteristics of the sex-stratified study population are presented in Table 1. The total number of participants was 206,719, of which 91,805 were men, and 114,914 were women. Overall, 66,390 (72.3%) of the 91,805 men and 90,297 (78.6%) of the 114,914 women included in the study were considered to have poor sleep quality. Among them, 12,005 (18.1%) of the 66,390 men and 22,227 (24.6%) of the 90,297 women were considered as experiencing SCD. A greater percentage of individuals with poor sleep quality exhibited cognitive decline compared to that of those with good sleep quality. Further, the percentages of men with SCD were 24.7, 17.7, 13.3, 12.9, 15.0, and 21.8% in the < 5, 5 ≤ to < 6, 6 ≤ to < 7, 7 ≤ to < 8, 8 ≤ to < 9, and 9 ≤ h groups, respectively, while the proportions of women with SCD were 32.7, 25.3, 20.4, 17.7, 17.7, and 23.1% in the < 5, 5 ≤ to < 6, 6 ≤ to < 7, 7 ≤ to < 8, 8 ≤ to < 9, and 9 ≤ h groups, respectively.

**Table 1:** General characteristics of the study population.

Variables	Subjective cognitive decline											
	Men (n = 91,805)						Women (n = 114,914)					
	Total	No SCD		SCD		P-value*	Total	No SCD		SCD		P-value*
	N	N	%	N	%		N	N	%	N	%	
**Sleep quality (PSQI)**^**a**^						< .0001						< .0001
Good	25,415	23,517	92.5	1,898	7.5		24,617	22,036	89.5	2,581	10.5	
Poor	66,390	54,385	81.9	12,005	18.1		90,297	68,070	75.4	22,227	24.6	
**Sleep duration (hour)**						< .0001						< .0001
< 5	6,607	4,973	75.3	1,634	24.7		12,561	8,453	67.3	4,108	32.7	
5 ≤ to < 6	14,371	11,824	82.3	2,547	17.7		20,119	15,028	74.7	5,091	25.3	
6 ≤ to < 7	28,533	24,732	86.7	3,801	13.3		33,381	26,572	79.6	6,809	20.4	
7 ≤ to < 8	28,476	24,789	87.1	3,687	12.9		32,054	26,384	82.3	5,670	17.7	
8 ≤ to < 9	11,433	9,719	85.0	1,714	15.0		13,828	11,384	82.3	2,444	17.7	
9 ≤	2,385	1,865	78.2	520	21.8		2,971	2,285	76.9	686	23.1	
**Age (year)**^**b**^	53.5	47.5	± 17.8	55.8	± 16.5	< .0001	55.3	47.0	± 17.4	57.6	± 17.2	< .0001
**Marital status**						< .0001						< .0001
Married	66,260	55,211	83.3	11,049	16.7		72,417	57,556	79.5	14,861	20.5	
Divorced, separated or widowed	7,974	6,152	77.2	1,822	22.8		28,823	19,840	68.8	8,983	31.2	
Unmarried	17,571	16,539	94.1	1,032	5.9		13,674	12,710	93.0	964	7.0	
**Educational level**						< .0001						< .0001
High school or below	53,680	43,223	80.5	10,457	19.5		79,414	58,844	74.1	20,570	25.9	
College	34,160	31,141	91.2	3,019	8.8		32,853	28,970	88.2	3,883	11.8	
Graduate school or above	3,965	3,538	89.2	427	10.8		2,647	2,292	86.6	355	13.4	
**Region**						< .0001						< .0001
Metropolitan	25,696	22,431	87.3	3,265	12.7		31,434	25,445	80.9	5,989	19.1	
Urban	16,555	14,127	85.3	2,428	14.7		20,603	16,288	79.1	4,315	20.9	
Rural	49,554	41,344	83.4	8,210	16.6		62,877	48,373	76.9	14,504	23.1	
**Household income**						< .0001						< .0001
Low	19,316	14,281	73.9	5,035	26.1		32,602	22,539	69.1	10,063	30.9	
Mid-low	21,548	17,968	83.4	3,580	16.6		24,984	19,513	78.1	5,471	21.9	
Mid-high	25,023	22,183	88.7	2,840	11.3		27,845	23,032	82.7	4,813	17.3	
High	25,918	23,470	90.6	2,448	9.4		29,483	25,022	84.9	4,461	15.1	
**Job**						< .0001						< .0001
Specialized job	10,922	9,943	91.0	979	9.0		10,338	9,123	88.2	1,215	11.8	
Office worker	9,638	8,899	92.3	739	7.7		8,947	7,916	88.5	1,031	11.5	
Sales and service	9,037	8,095	89.6	942	10.4		16,485	13,588	82.4	2,897	17.6	
Agriculture and fishery	12,807	10,086	78.8	2,721	21.2		10,489	7,760	74.0	2,729	26.0	
Manual worker	25,164	22,096	87.8	3,068	12.2		13,083	10,310	78.8	2,773	21.2	
Others^c^	24,237	18,783	77.5	5,454	22.5		55,572	41,409	74.5	14,163	25.5	
**Depressive symptom (PHQ-9)**						< .0001						< .0001
Yes	2,541	1,262	49.7	1,279	50.3		5,427	2,501	46.1	2,926	53.9	
No	89,264	76,640	85.9	12,624	14.1		109,487	87,605	80.0	21,882	20.0	
**Stress**						< .0001						< .0001
Yes	20,742	16,718	80.6	4,024	19.4		27,719	19,889	71.8	7,830	28.2	
No	71,063	61,184	86.1	9,879	13.9		87,195	70,217	80.5	16,978	19.5	
**Health-related behaviors**^**d**^						0.6786						< .0001
Yes	23,912	20,271	84.8	3,641	15.2		46,503	37,348	80.3	9,155	19.7	
No	67,893	57,631	84.9	10,262	15.1		68,411	52,758	77.1	15,653	22.9	
**BMI (kg/m**^**2**^**)**^**e**^						< .0001						< .0001
Underweight or normal	33,220	28,829	86.8	4,391	13.2		28,062	21,699	77.3	6,363	22.7	
Overweight	24,772	21,139	85.3	3,633	14.7		23,136	18,091	78.2	5,045	21.8	
Obese	33,813	27,934	82.6	5,879	17.4		63,716	50,316	79.0	13,400	21.0	
**Total**	91,805	77,902	84.9	13,903	15.1		114,914	90,106	78.4	24,808	21.6	

*PSQI* Pittsburgh Sleep Quality Index, *SCD* subjective cognitive decline, *CI* confidence interval, *OR* odds ratio, *PHQ-9* Patient Health Questionnaire-9.

^a^Sleep quality: Sleep quality was measured using the Korean version of Pittsburgh Sleep Quality Index (PSQI-K). Good sleep quality ≤ 5, poor sleep quality > 5.

^b^Values are presented as mean ± standard deviation.

^c^Others: Others include students, housewives, the unemployed and exclude occupational soldiers.

^d^Health-related behaviors: Health-related behaviors include not currently smoking, not high risk alcohol use, and engaging in walking physical activity more than 30 min per day for more than 5 days in the last week.

^e^BMI: body mass index; Obesity status defined by BMI based on 2014 Clinical Practice Guidelines for Overweight and Obesity in Korea.

*P-values were obtained by t-test or Chi-square test.

Table 2 presents the logistic regression analysis results of both men and women adjusted for all the covariates. Individuals with poor sleep quality were more likely to experience SCD in the case of both men and women. Compared with those of the reference group (good sleep quality), the ORs (95% CIs) for cognitive decline in the group with poor sleep quality were as follows: OR = 1.89 [95% CI 1.75–2.04] in men and OR = 1.74 [95% CI 1.63–1.85] in women. Additionally, our study identified that sleep duration was significantly associated with cognitive decline. Our analysis revealed a U-shaped pattern between sleep duration and cognitive decline. The ORs were significant in all the groups for both the sexes except for in the group of men with a sleep duration of 6 ≤ to < 7 h. In both sexes, the group that overslept for 9 h or longer was the most likely one to experience SCD.

**Table 2:** Odds ratio for subjective cognitive decline.

Variables	Subjective cognitive decline					
	Total		Men		Women	
	Adjusted OR	95% CI	Adjusted OR	95% CI	Adjusted OR	95% CI
**Sleep quality (PSQI)**^**a**^						
Good	1.00		1.00		1.00	
Poor	1.81	(1.72–1.90)	1.89	(1.75–2.04)	1.74	(1.63–1.85)
**Sleep duration**						
< 5	1.40	(1.33–1.49)	1.41	(1.28–1.55)	1.41	(1.32–1.51)
5 ≤ to < 6	1.19	(1.13–1.25)	1.19	(1.10–1.29)	1.20	(1.13–1.27)
6 ≤ to < 7	1.07	(1.03–1.12)	1.01	(0.94–1.08)	1.12	(1.06–1.19)
7 ≤ to < 8	1.00		1.00		1.00	
8 ≤ to < 9	1.15	(1.09–1.22)	1.26	(1.15–1.38)	1.07	(0.99–1.15)
9 ≤	1.41	(1.28–1.56)	1.37	(1.18–1.60)	1.40	(1.22–1.60)
**Age**	1.04	(1.03–1.04)	1.04	(1.04–1.05)	1.03	(1.03–1.03)
**Marital status**						
Married	1.00		1.00		1.00	
Divorced, separated or widowed	0.98	(0.93–1.02)	1.01	(0.93–1.10)	1.03	(0.98–1.09)
Unmarried	0.76	(0.71–0.82)	0.95	(0.85–1.06)	0.64	(0.59–0.70)
**Educational level**						
High school or below	1.01	(0.90–1.12)	1.04	(0.90–1.19)	0.95	(0.81–1.10)
College or university	0.92	(0.83–1.02)	0.98	(0.85–1.12)	0.84	(0.73–0.98)
Graduate school or above	1.00		1.00		1.00	
**Region**						
Metropolitan	0.97	(0.93–1.01)	0.98	(0.92–1.04)	0.95	(0.91–1.00)
Urban	1.02	(0.97–1.07)	1.05	(0.98–1.13)	0.99	(0.94–1.05)
Rural	1.00		1.00		1.00	
**Household income**						
Low	1.07	(1.00–1.13)	1.10	(1.00–1.21)	1.03	(0.96–1.10)
Mid–low	1.07	(1.02–1.13)	1.08	(0.99–1.18)	1.04	(0.98–1.11)
Mid–high	1.04	(0.99–1.10)	1.06	(0.98–1.15)	1.03	(0.97–1.09)
High	1.00		1.00		1.00	
**Job**						
Specialized job	1.00		1.00		1.00	
Office worker	1.01	(0.94–1.10)	0.99	(0.88–1.12)	1.05	(0.94–1.16)
Sales and service	1.20	(1.12–1.30)	1.23	(1.09–1.40)	1.15	(1.04–1.26)
Agriculture and fishery	1.20	(1.11–1.30)	1.29	(1.15–1.44)	1.08	(0.97–1.20)
Manual worker	1.08	(1.01–1.17)	1.10	(0.99–1.22)	1.09	(0.99–1.20)
Others^b^	1.27	(1.19–1.36)	1.32	(1.19–1.47)	1.19	(1.09–1.29)
**Depressive symptom (PHQ**-**9)**						
Yes	3.38	(3.14–3.64)	4.09	(3.58–4.66)	3.02	(2.77–3.29)
No	1.00		1.00		1.00	
**Stress**						
Yes	1.63	(1.57–1.69)	1.69	(1.59–1.80)	1.61	(1.53–1.69)
No	1.00		1.00		1.00	
**Health**-**related behaviors**^**c**^						
Yes	1.00		1.00		1.00	
No	1.09	(1.05–1.12)	1.08	(1.02–1.15)	1.11	(1.06–1.15)
**BMI (kg/m**^**2**^**)**^**d**^						
Underweight or normal	1.00		1.00		1.00	
Overweight	1.00	(0.96–1.04)	0.95	(0.89–1.01)	1.02	(0.96–1.08)
Obese	1.02	(0.98–1.05)	0.99	(0.93–1.05)	0.99	(0.94–1.04)

*PSQI* Pittsburgh Sleep Quality Index, *CI* confidence interval, *OR* odds ratio, *PHQ-9* Patient Health Questionnaire-9.

^a^Sleep quality: Sleep quality was measured using the Korean version of Pittsburgh Sleep Quality Index (PSQI**-**K). Good sleep quality ≤ 5, poor sleep quality > 5.

^b^Others: Others include students, housewives, the unemployed and exclude occupational soldiers.

^c^Health-related behaviors: Health-related behaviors include not currently smoking, not high risk alcohol use, and engaging in walking physical activity more than 30 min per day for more than 5 days in the last week.

^d^BMI: body mass index; Obesity status defined by BMI based on 2014 Clinical Practice Guidelines for Overweight and Obesity in Korea.

In order to analyze the relationship between each component of the PSQI and SCD, all the independent variables were adjusted for, and a multiple logistic regression analysis was performed (Table 3). Among the responses to each component item, the best response was considered as 0, and individuals with this response were set as the reference group in the analysis. We observed a dose–response relationship between the subjective sleep quality, sleep disturbance, and daytime dysfunction components and SCD in both men and women. In men, there was also a dose–response relationship between the component for the use of sleep medication and SCD. The ORs were significant in all the groups.

**Table 3:** The results of the analysis on the association between each component of the PSQI and subjective cognitive decline.

Variables	Subjective cognitive decline			
	Men		Women	
	Adjusted OR*	95% CI	Adjusted OR*	95% CI
**Subjective sleep quality**				
Very good	1.00		1.00	
Fairy good	1.16	(1.07–1.26)	1.11	(1.04–1.19)
Fairly bad	1.96	(1.78–2.15)	1.68	(1.55–1.81)
Very bad	2.59	(2.19–3.06)	1.96	(1.75–2.19)
**Sleep latency (min)**				
≤ 15	1.00		1.00	
16–30	1.20	(1.13–1.27)	1.14	(1.09–1.20)
31–60	1.67	(1.54–1.81)	1.49	(1.40–1.58)
> 60	1.86	(1.70–2.04)	1.57	(1.48–1.68)
**Sleep duration (h)**				
> 7	1.00		1.00	
6–7	0.96	(0.81–1.14)	1.06	(0.93–1.21)
5–6	0.86	(0.69–1.07)	1.05	(0.89–1.25)
< 5	0.88	(0.67–1.16)	0.99	(0.79–1.23)
**Habitual sleep efficiency (%)**				
≥ 85	1.00		1.00	
75–84	1.13	(0.93–1.38)	1.18	(1.03–1.35)
65–74	1.16	(0.93–1.46)	0.94	(0.79–1.11)
< 65	0.91	(0.84–0.99)	0.94	(0.89–0.99)
**Sleep disturbance**				
0	1.00		1.00	
1–9	2.32	(2.12–2.54)	2.22	(2.06–2.40)
10–18	5.04	(4.47–5.69)	4.50	(4.10–4.93)
19–27	6.60	(4.38–9.95)	5.40	(4.14–7.05)
**Use of sleep medication**				
Not during the past ,onth	1.00		1.00	
less than once a week	1.75	(1.38–2.24)	1.51	(1.30–1.74)
Once or twice a week	1.73	(1.36–2.20)	1.52	(1.27–1.82)
Three or more times a week	1.98	(1.66–2.36)	1.53	(1.37–1.70)
**Daytime dysfunction**				
No problem at all	1.00		1.00	
only a very slight problem	1.99	(1.87–2.12)	1.76	(1.68–1.85)
Somewhat of a problem	3.02	(2.79–3.28)	2.61	(2.45–2.77)
A very big problem	5.17	(4.35–6.14)	3.56	(3.14–4.03)

*PSQI* Pittsburgh Sleep Quality Index, *CI* confidence interval, *OR* odds ratio.

*OR adjusted for all covariates considered in the study.

**Figure 1:**
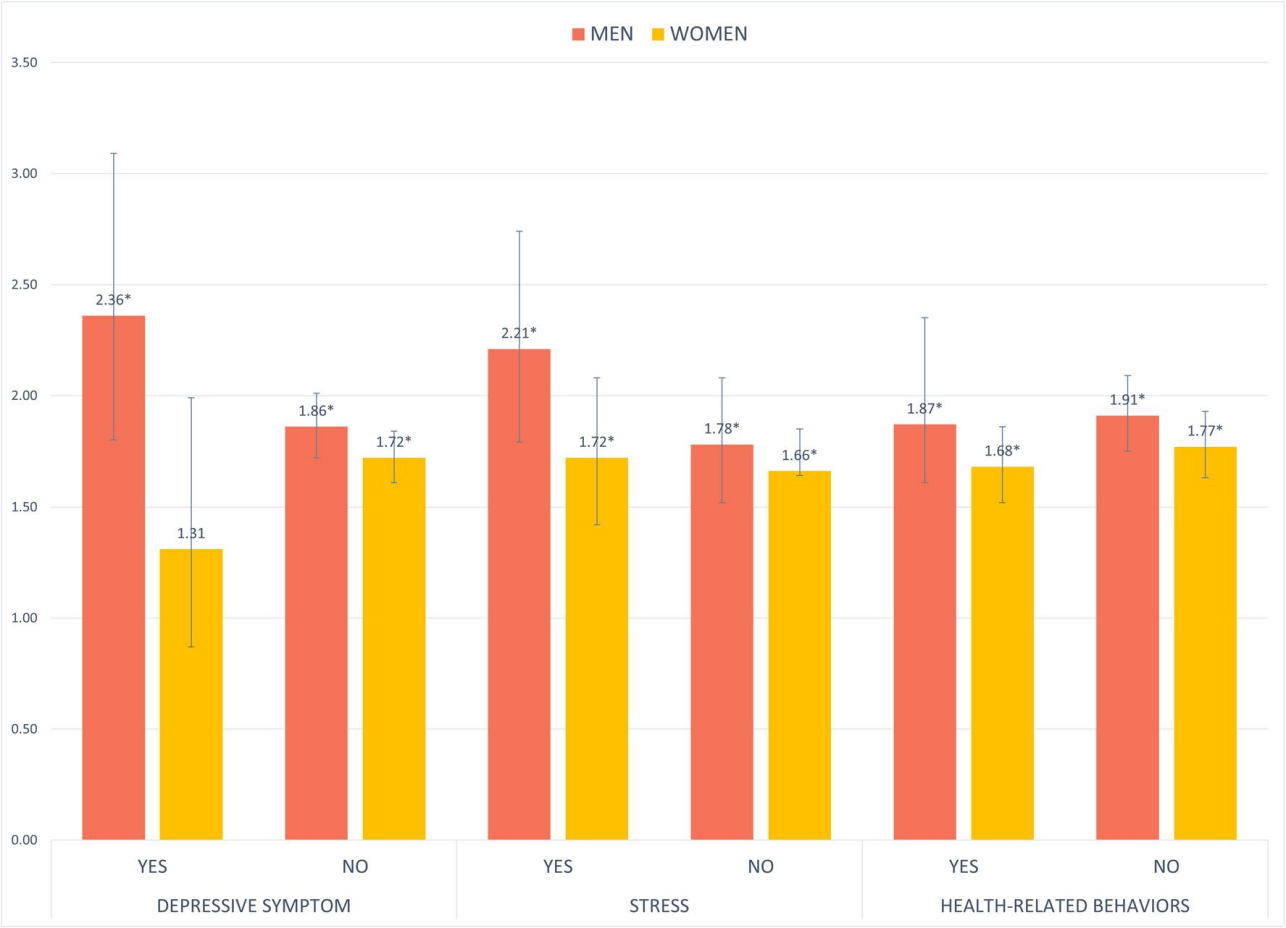
The results of subgroup analysis for the association between sleep quality and subjective cognitive decline. ^†^Analysis was adjusted for all covariates in the study. ^‡*^indicate statistically significant results (*P* < .05).

Figure 1 (eTable 1) outlines the results of the subgroup analysis regarding the effects of depression, stress, and health-related behaviors on SCD according to sleep quality. In men with poor sleep quality, individuals with depressive symptom (OR = 2.47 [95% CI 1.56–3.90]) were more likely to experience SCD compared to individuals without depressive symptom (OR = 2.36 [95% CI 1.80–3.09]). The same results were found in the case of those with stress (OR = 2.21 [95% CI 1.79–2.74] for men; OR = 1.72 [95% CI 1.42–2.08] for women) in comparison to those without stress. Both men and women who did not engage in beneficial health-related behaviors were found to be more likely to exhibit a decline in cognitive function if their sleep quality was poor compared to those with good health-related behaviors (OR = 1.91 [95% CI 1.75–2.09] for men; OR = 1.77 [95% CI 1.63–1.93] for women).

## Discussion

Sleep, an essential element of everyday life, is closely related to health. It is widely believed that poor sleep behaviors have a negative effect on an individual’s general health. This study was conducted to identify the association between sleep quality and duration and SCD in the Korean population using representative data recorded via the KCHS. We also conducted a subgroup analysis based on depression, perceived stress, and health-related behaviors, which are factors related to sleep patterns.

We observed that individuals who had poor sleep quality and short or long sleep durations were more likely to exhibit SCD compared to those who had good sleep quality and an adequate sleep duration. Odds ratios for men and women with poor sleep quality were 1.97 and 1.75 more likely to experience SCD, respectively. Additionally, in both sexes, the likelihood of developing cognitive impairment increased in individuals with insufficient or excessive sleep and not in those with an adequate sleep duration. The findings of the present study were consistent with those of earlier studies, which suggests that poor sleep is associated with cognitive function^17,26,27^. Another study conducted on Koreans also revealed that poor sleep quality can cause cognitive and functional decline^17^.

Several studies have demonstrated the effect of sleep quality on cognitive decline. A previous study has shown that poor sleep quality is associated with amyloid deposition^26^. Amyloid can affect the Alzheimer disease pathogenesis^28^. Also, sleep problems can cause more of the stress hormone cortisol, which can lead to brain inflammation and tissue damage^29^. In several cross-sectional studies, insufficient or decreased sleep quality was found to be associated with poor cognitive function^30–32^. In addition, prior cross-sectional study of older adults has shown that a nighttime sleep duration of 6 h or less and daytime sleep duration of 1 h or more could be associated with poor cognitive impairment with daytime sleepiness^33^.

Prior studies have also reported on a link between sleep duration and cognitive decline^34^. In a study that examined the association between sleep related factors and memory impairment in older Chinese individuals, an inverted U-shaped association was found between sleep duration and scores on the word delayed test, with the peak occurring at 7 to 8 h^31^. However, another study reported that sleep quality is more relevant to cognitive decline than sleep duration. The results of research conducted on elderly women suggest that the disturbance of sleep rather than quantity could be likelihood of poorer cognition. Sleep disturbances, such as waking up an increasing number of times after sleep onset, lower sleep efficiency, and lower sleep latency, were found to be consistently related to poorer cognition levels whereas total sleep time was not^30^. Another prospective study demonstrated that obstructive sleep apnea, a common sleep disorder that causes sleep disruption and hypoxia, increased the prospective risk of dementia in a cohort of elderly women^35^.

In addition, there are some studies that have demonstrated that sleep is associated with health or mental health outcomes, such as stress^8,9,36–38^. An epidemiological study that included over one million American subjects reported that under 5 h or 10 h or more of sleep were predictors of mortality that were almost as strong as “ever had” diabetes, heart disease, stroke, or high blood pressure. The increased mortality rates were seen in both sexes and across all age groups^10^.

Previous study has shown that there is a greater association between poor health-related behaviors, such as smoking and high-risk drinking, and poor sleep quality in women^18^. In terms of psychiatry, subjective health status, stress, depressive symptoms, and subjective cognitive decline were strongly associated with poor sleep quality in both men and women. Some studies found that sleep disturbances were more relevant in subjects with poor mental health^39,40^. The result of this study was consistent with previous studies indicating that depressive symptoms were associated with cognitive decline^41,42^.

This study has several limitations that should be considered when interpreting the results. First, as this study used cross-sectional data, a clear causal relationship between the quality and duration of sleep and cognitive function could not be inferred. Second, because sleep quality and average sleep duration were evaluated based on the respondent’s memory, there is a likelihood of recall bias. Third, cognitive decline was evaluated using subjective measurement in a self-report format and not a clinical diagnosis or experimental test. Therefore, this study has a limitation in that it did not objectively measure cognitive performance with appropriate tests. Fourth, it was not possible to consider unmeasured confounders, such as clinical sleep disorders or caffeine use, in this study.

Despite these limitations, the present study has some strengths. First, we used nationwide representative data that are suitable for conducting Korean studies. The study subjects were randomly sampled from community-based populations. Second, the study was based on the first nationwide survey that used the PSQI to measure sleep quality in Korea. The PSQI is a highly validated and reliable index of sleep quality, which is used worldwide. Third, mental health-related confounders, including depression and stress, which are strongly associated with sleep were included in the analysis. Furthermore, this study is meaningful in that its results support and add to the findings of previous studies by demonstrating that good sleep quality and an adequate sleep duration can help in preventing cognitive impairment. Thus, we could suggest that public policy and education developed for the promotion of mental health in Koreans should be focused on good sleep behaviors.

## Conclusion

This study demonstrated that poor sleep behaviors are negatively associated with cognitive function in the Korean population. In addition, depression, stress, and bad health-related behaviors tend to increase the risk of cognitive impairment. These findings indicate that the potential benefits of good sleep quality and an optimal sleep duration could effectively prevent cognitive decline. Therefore, our research could help health policymakers and professionals in recognizing sleep behaviors as risk factors for a decline in cognitive function and in implementing prevention and intervention strategies for managing public mental health by promoting the benefits of good sleep quality and an optimal sleep duration.

## Supplementary Information

Supplementary Information.

## Data Availability

The data of Korea Community Health Survey (KCHS) are publicly available through the Community Health Survey website (https://chs.cdc.go.kr).
