# Supplementary material for: Association between quality and duration of sleep and subjective cognitive decline: a cross-sectional study in South Korea
Source: Sci Rep. 2021 Aug 20;11:16989. doi: 10.1038/s41598-021-96453-x (PMC8379242; doi:10.1038/s41598-021-96453-x)
Supplement: Supplementary file 1 — Supplementary Information. [file 41598_2021_96453_MOESM1_ESM.docx]

**Online supplements**

**eTable 1. The results of subgroup analysis for the association between sleep quality and subjective cognitive decline**

| **eTable 1. The results of subgroup analysis for the association between sleep quality and subjective cognitive decline** | | | | | | | | | |
| --- | --- | --- | --- | --- | --- | --- | --- | --- | --- |
| **Subgroups** | **Sleep quality (PSQI)^a^** | **Subjective cognitive decline** | | | | | | | |
|  |  | **Men** | | | | **Women** | | | |
|  |  | **Adjusted OR^*^** | **95% CI** | | | **Adjusted OR^*^** | **95% CI** | | |
| **Depressive symptom (PHQ-9)** |  |  |  |  |  |  |  |  |  |
| Yes | Good | 1.00 |  |  |  | 1.00 |  |  |  |
|  | Poor | 2.36 | (1.80 | - | 3.09) | 1.31 | (0.87 | - | 1.99) |
| No | Good | 1.00 |  |  |  | 1.00 |  |  |  |
|  | Poor | 1.86 | (1.72 | - | 2.01) | 1.72 | (1.61 | - | 1.84) |
| **Stress** |  |  |  |  |  |  |  |  |  |
| Yes | Good | 1.00 |  |  |  | 1.00 |  |  |  |
|  | Poor | 2.21 | (1.79 | - | 2.74) | 1.72 | (1.42 | - | 2.08) |
| No | Good | 1.00 |  |  |  | 1.00 |  |  |  |
|  | Poor | 1.78 | (1.52 | - | 2.08) | 1.66 | (1.48 | - | 1.85) |
| **Health-related behaviors^b^** |  |  |  |  |  |  |  |  |  |
| Yes | Good | 1.00 |  |  |  | 1.00 |  |  |  |
|  | Poor | 1.87 | (1.61 | - | 2.16) | 1.68 | (1.52 | - | 1.86) |
| No | Good | 1.00 |  |  |  | 1.00 |  |  |  |
|  | Poor | 1.91 | (1.75 | - | 2.09) | 1.77 | (1.63 | - | 1.93) |
| ^a^Sleep quality: Sleep quality was measured using the Korean version of Pittsburgh Sleep Quality Index (PSQI-K). Good sleep quality ≤ 5, poor sleep quality > 5. | | | | | | | | | |
| ^b^Health-related behaviors: Health-related behaviors include not currently smoking, not high risk alcohol use, and engaging in walking physical activity more than 30 min per day for more than 5 days in the last week. | | | | | | | | | |
| ^*^OR adjusted for all covariates considered in the study | | | | | | | | | |
| Abbreviations: PSQI, Pittsburgh Sleep Quality Index; CI, confidence interval; OR, odds ratio; PHQ-9, Patient Health Questionnaire-9 | | | | | | | | | |
